# Entecavir vs Tenofovir in Hepatocellular Carcinoma Prevention in Chronic Hepatitis B Infection: A Systematic Review and Meta-Analysis

**DOI:** 10.14309/ctg.0000000000000236

**Published:** 2020-10-06

**Authors:** Ka Shing Cheung, Lung Yi Mak, Sze Hang Liu, Ho Ming Cheng, Wai Kay Seto, Man Fung Yuen, Ching Lung Lai

**Affiliations:** 1 Department of Medicine, The University of Hong Kong, Queen Mary Hospital, Hong Kong;; 2 Department of Medicine, The University of Hong Kong-Shenzhen Hospital, Shenzhen, China.

## Abstract

Entecavir (ETV) and tenofovir disoproxil fumarate (TDF) are recommended as first-line therapies for chronic hepatitis B (CHB) infection. Although both drugs reduce hepatocellular carcinoma (HCC) risk, their comparative effectiveness remains controversial. We aimed to determine whether TDF is superior to ETV in preventing HCC.

**METHODS:**

PubMed, Embase, and Cochrane Library from inception until June 9, 2020, were searched according to the Preferred Reporting Items for Systematic Reviews and Meta-Analyses guidelines. Key terms included entecavir, tenofovir, and hepatocellular carcinoma. The adjusted hazard ratios (HRs) were pooled using a random effects model. Heterogeneity among studies was assessed by the Cochran *Q* test and *I*^2^.

**RESULTS:**

Thirteen observational studies (4 of which were conference abstracts) were included with 85,008 patients with CHB (ETV: 56,346; TDF: 28,662). TDF was associated with a lower HCC risk (adjusted HR [aHR]: 0.81; 95% confidence interval [CI]: 0.67–0.99). This beneficial effect was present in cirrhotic patients (aHR: 0.73; 95% CI: 0.62–0.85) and retrospective cohort studies using electronic data sets (aHR: 0.63; 95% CI: 0.51–0.78). However, this beneficial effect did not reach statistical significance for noncirrhotic patients (aHR: 0.83, 95% CI: 0.51–1.35) and retrospective/prospective cohort studies using clinical records (aHR: 0.97; 95% CI: 0.80–1.18).

**DISCUSSION:**

TDF was associated with a lower HCC risk compared with ETV among patients with CHB, particularly cirrhotic patients. Further prospective large-scale studies with longer follow-up periods were required to identify specific subgroups that will benefit most from TDF.

## INTRODUCTION

Chronic hepatitis B (CHB) virus infection is a global public health threat, with 257 million people being affected worldwide in 2016. It can lead to chronic hepatitis, hepatitic flare up, cirrhosis, hepatocellular carcinoma (HCC), and mortality (1–3). Nucleos(t)ide analogs (NAs) can reduce HBV-related cirrhosis and its associated complications (4,5). Both entecavir (ETV) and tenofovir (tenofovir disoproxil fumarate [TDF] and tenofovir alafenamide) are recommended first-line therapies for the treatment of CHB (6) because they have high antiviral potency and a high genetic barrier to resistance (4). Most comparative studies have been performed comparing ETV with TDF because tenofovir alafenamide is licensed relatively recently.

TDF is more effective in viral suppression, especially in hepatitis B e-antigen (HBeAg)-positive patients with very high HBV DNA (7,8), but is associated with risks of renal impairment and hypophosphatemia (9). Although both ETV and TDF reduce HCC risk (4,10), there are no randomized clinical trials (RCTs) directly comparing their effects. Despite profound viral suppression with undetectable serum HBV DNA under NAs, HCC can still develop (11). The nationwide study by Choi et al. (12) that showed a better HCC preventive effect of TDF over ETV has recently sparked much interest in this topic. Subsequent studies, however, yielded conflicting results, with some showing concordant results (13,14), whereas others showing no difference between these 2 NAs (15–24). Although most of these studies were of high quality and were nationwide or multicenter studies using propensity score (PS) matching to minimize selection bias and confounding factors, controversy arises from because of (i) inadequate sample size of some studies, (ii) different ethnicities (Asian vs non-Asian), (iii) different proportions of cirrhotic patients across studies, and (iv) study design with different natures of the data sets (with electronic/claims databases more likely to suffer from residual confounding despite PS methodology).

Two recent meta-analyses did not include most of the recent high-quality studies (25,26). In addition, most of the included studies did not investigate HCC as the primary outcome, and hence, only the number of events or unadjusted effect estimates were reported. Significant differences in baseline characteristics could exist between patients receiving ETV and TDF because of indication/selection bias. The pooling of unadjusted odds ratio (instead of adjusted hazard ratio [aHR]) will therefore suffer from serious bias, by favoring TDF over ETV, in these 2 meta-analyses. Instead, the use of HR gives a less biased estimate by taking the time element (follow-up duration) into consideration.

We therefore aimed to compare the effect of ETV and TDF in reducing HCC risk by including the more recent high-quality studies, with a detailed subgroup analysis to explain how the conflicting results among various studies could arise.

## METHODS

### Study selection

Three databases including PubMed, Embase, and Cochrane Library were searched following the Preferred Reporting Items for Systematic Reviews and Meta-Analyses guideline (27) from inception until June 9, 2020. All the tenofovir comparison data retrieved were TDF only. The search details are shown in the supplementary file (see Supplementary Digital Content 1, http://links.lww.com/CTG/A384). Potential studies were retrieved after title/abstract screening by the investigator (K.S.C.). All articles were imported to Endnote X9.2 (Thompson and Reuters, Philadelphia, Pennsylvania), and duplicates were removed.

### Selection criteria

Two authors (K.S.C. and L.Y.M.) determined the eligibility of studies independently, and dissonance was resolved by 2 senior authors (M.F.Y. and C.L.L.). The inclusion criteria included the following: (i) study population: patients with CHB; (ii) treatment: ETV vs tenofovir monotherapy; (iii) study design: prospective/retrospective cohort study, case-control study, and RCT; and (iv) outcome: HCC. There was no language restriction.

The exclusion criteria were (i) hepatitis C or D virus, or human immunodeficiency virus coinfection; (ii) previous HCC or liver transplantation; (iii) studies that did not report adjusted effect estimate (by either PS methodology or multivariable analysis); and (iv) review articles, meta-analyses, editorials, case reports, and other forms (e.g., commentary and letters to the editors).

Studies that did not report adjusted effect estimates were excluded because marked imbalance in baseline characteristics exists between ETV and TDF. As ETV was introduced to the market much earlier with a longer available data of drug efficacy and safety than TDF, it may be preferentially given to patients with more severe liver disease. In addition, patients with underlying renal impairment or at risk of renal impairment/osteoporosis (e.g., elderly, diabetes, and hypertension) tend to receive ETV because of TDF-associated renal toxicity. If this indication/selection bias is not taken into consideration by adjusting for liver disease severity, comorbidities, and age, the association could be biased toward favoring TDF in HCC risk reduction.

### Data extraction and quality assessment

For the eligible articles, we recorded items including first authors, publication year, ethnicity, study design, inclusion/exclusion criteria, exposure of interest, outcome of interest, sample size, age, sex, HBeAg, cirrhosis, alanine aminotransferase (ALT), HBV DNA, previous NA/pegylated-interferon use, follow-up time, and variables taken into consideration in PS matching or multivariable analysis. The PS represents probability of prescribing TDF instead of ETV that is dependent on other covariates (e.g., age, sex, HBeAg, cirrhosis, ALT, and HBV DNA) (28). PS matching ensures balance of measured confounding factors between ETV and TDF groups so that any difference in the outcome (HCC) would ideally be due to the effect of NA use only.

We also recorded data of HR derived by the PS matching or PS regression adjustment/multivariable analysis. Two authors (K.S.C. and L.Y.M.) assessed the quality of observational studies by the Newcastle-Ottawa scale independently (29). Authors were contacted for more information if deemed necessary.

### Data analysis

All statistical analyses were performed using R version 3.2.3 (R Foundation for Statistical Computing) statistical software. Continuous variables were expressed as median (interquartile range [IQR]) or mean (±1 SD). Comparisons of outcome were expressed as HR and 95% confidence interval (CI) using the random effects model and were presented as Forest plot. We used the Cochran *Q* test to detect heterogeneity among studies, with a *P* value <0.1 indicating significant heterogeneity. We calculate *I*^2^ statistic to measure the proportion of total variation in the study estimates attributed to heterogeneity. *I*^2^ values of <25%, 25%–75%, and >75% indicate low, moderate, and high heterogeneities, respectively (30). Publication bias across studies was assessed by visual inspection of funnel plots and Egger linear regression tests (31).

Eight studies reported aHR derived by both PS matching and multivariable analysis, whereas 5 reported aHR by multivariable analysis only.

The pooled HR in the current meta-analysis was calculated by pooling individual HR derived by PS matching in various studies. However, if a particular study had not reported HR by PS matching, the aHR by multivariable analysis would be pooled into the analysis. PS matching is preferred to multivariable analysis because PS matching achieves good balance of baseline characteristics between the 2 groups to minimize bias by its quasiexperimental design.

A subgroup analysis was performed according to cirrhosis, ethnicity (Asian vs non-Asian), and study design (prospective/retrospective cohort studies using clinical records vs retrospective cohort studies using electronic data sets).

Sensitivity analyses were performed by (i) leave-one-out analysis (in which the pooled aHR was recalculated by excluding 1 study at a time) to assess the impact of a single study on the pooled effect estimate. In particular, subgroup analyses were also performed by excluding the study by Choi et al. because it accounted for the largest weight in the pooled analysis; (ii) pooling aHR derived by multivariable analysis instead of PS matching in various studies; (iii) excluding studies that recruited treatment-experienced subjects; and (iv) using fixed effects model.

## RESULTS

### Study and patient characteristics

**Figure 1.**
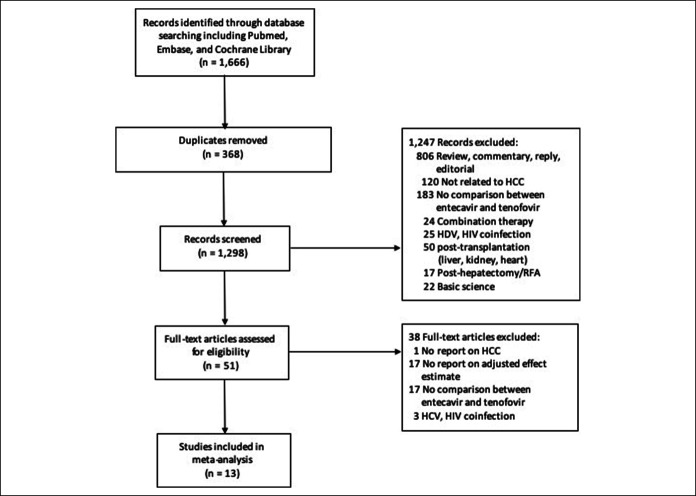
Study selection flow diagram. HCC, hepatocellular carcinoma; HDV, hepatitis D virus; RFA, radiofrequency ablation.

Figure 1 depicts the study selection process. Thirteen of 1,666 studies remained for meta-analysis, with 4 being conference abstracts. All included studies were observational studies (Table 1) and scored 8–9 stars in the Newcastle-Ottawa scale indicating satisfactory quality (see eTable 1, Supplementary Digital Content 1, http://links.lww.com/CTG/A384).

**Table 1.**
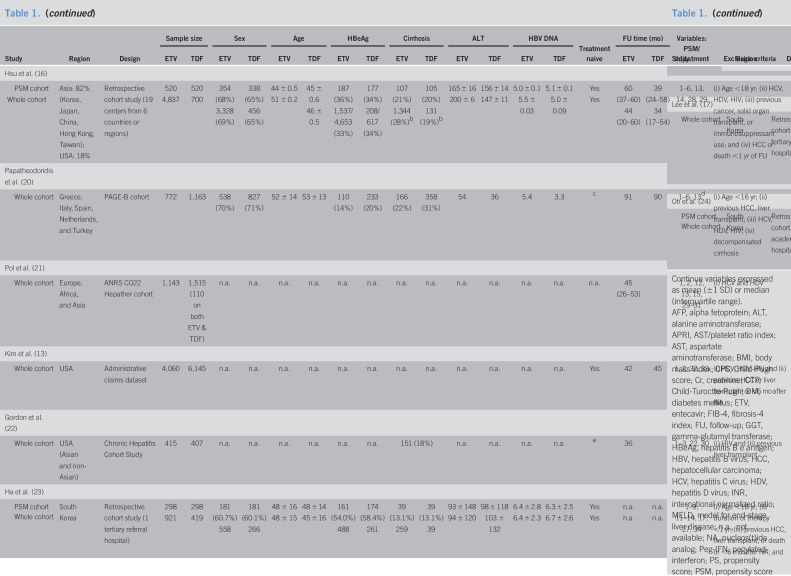

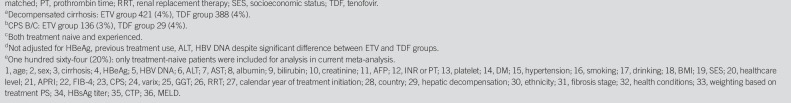
Baseline characteristics of the included studies (n = 13)

Study	Region	Design	Sample size		Sex		Age		HBeAg		Cirrhosis		ALT		HBV DNA		Treatment naive	FU time (mo)		Variables: PSM/adjustment	Exclusion criteria
			ETV	TDF	ETV	TDF	ETV	TDF	ETV	TDF	ETV	TDF	ETV	TDF	ETV	TDF		ETV	TDF		
Kim et al. (15)																					
PSM cohort Whole cohort	South Korea	Retrospective cohort study (1 tertiary referral hospital)	354 721	354 604	220 (62%) 471 (65%)	222 (63%) 363 (60%)	51 ± 12 52 ± 11	52 ± 11 50 ± 11	232 (66%) 430 (60%)	223 (64%) 376 (62%)	169 (48%) 346 (48%)	156 (44%) 267 (44%)	136 ± 159 143 ± 172	142 ± 229 137 ± 228	6.2 ± 1.4 6.4 ± 1.4	6.2 ± 1.5 6.0 ± 1.6	Yes Yes	n.a. 66 (36–88)	n.a. 33 (21–46)	1–15	(i) Decompensated cirrhosis, (ii) FU <1 yr, (iii) Cr >1.5 mg/dL, (iv) HBV DNA <2,000 IU/mL, and (v) death <6 mo or HCC <1 yr after NA
Choi et al. (12)																					
PSM cohort Whole cohort	South Korea	Retrospective cohort study (nationwide claims database of NHIS)	10,923 11,464	10,923 12,692	6,834 (62%) 11,464 (63%)	6,834 (63%) 12,692 (63%)	49 ± 10 49 ± 10	49 ± 10 49 ± 10	n.a. n.a.	n.a. n.a.	2,891 (27%)^a^ 2,991 (26%); D: 450 (4%)	2,919 (27%)^a^ 3,488 (28%); D: 414 (3%)	32 (21–54) 32 (21–54)	35 (24–57) 35 (24–58)	n.a. n.a.	n.a. n.a.	Yes Yes	51 (38–57) 51 (37–57)	37 (30–43) 37 (30–44)	1, 2, 3, 6, 14–20	(i) Age <30 or >80 yr; (ii) HCV, HDV, HIV; (iii) Previous organ transplant, HCC, or other cancer; and (iv) HCC, transplant, or death <6 mo after NA
Kim et al. (18)																					
PSM cohort Whole cohort	South Korea	Retrospective cohort study (4 academic teaching hospitals)	1,278 1,484	1,278 1,413	793 (62%) 889 (60%)	794 (62%) 913 (65%)	49 ± 11 48 ± 12	49 ± 12 49 ± 12	758 (50%) 758 (51%)	727 (50%) 694 (49%)	476 (32%) 499 (34%)	456 (32%) 411 (29%)	n.a. n.a.	n.a. n.a.	5.6 ± 2.1 5.7 ± 2.1	5.6 ± 2.1 5.4 ± 2.1	Yes Yes	n.a. 59	n.a.	1–4, 8, 9, 13–15	(i) Age <19 yr; (ii) decompensated cirrhosis; (iii) HCV, HDV; (iv) Previous organ transplant or HCC; (v) HCC, liver transplant, or death <6 mo of enrolment; (vi) and significant medical illness
Lee et al. (19)																					
PSM cohort Whole cohort	South Korea	Retrospective cohort study (1 tertiary referral hospital)	1,370 1,583	1,370 1,439	806 (59%) 926 (59%)	798 (58%) 841 (58%)	47 ± 12 47 ± 12	47 ± 11 47 ± 11	814 (59%) 974 (62%)	807 (59%) 823 (57%)	465 (34%) 567 (36%)	464 (34%) 483 (35%)	98 (53–200) 98 (53–201)	95 (50–196) 94 (51–194)	6.5 (5–8) 6.5 (5–8)	6.4 (5–8) 6.4 (5–8)	Yes Yes	n.a. 60	n.a. 36	1–15, 17, 18, 21–25	(i) HCV and HIV, (ii) HCC and transplant before or <6 mo after NA, (iii) other cancer, and (iv) decompensated cirrhosis
Yip et al. (14)																					
PSM cohort Whole cohort	Hong Kong, China	Retrospective cohort study (territory-wide healthcare database of public hospitals)	4,636 28,041	1,200 1,309	2,267 (49%) 18,094 (65%)	587 (49%) 591 (45%)	43 ± 13 53 ± 13	44 ± 13 43 ± 13	2,480 (54%) 8,317 (30%)	625 (52%) 721 (55%)	167 (4%) 3,822 (14%)	37 (3%) 38 (3%)	43 (25–108) 62 (33–137)	46 (26–107) 43 (25–103)	4.8 ± 2.8 5.3 ± 2.2	4.8 ± 2.7 4.9 ± 2.7	Yes Yes	35 (18–55) 44 (20–60)	34 (18–54) 34 (17–54)	1–6, 8–10, 12–15, 26–27	(i) HCV, HDV, HIV; (ii) autoimmune or metabolic liver disease; (iii) HCC and transplant before or <6 mo after NA; and (iv) FU <6 mo
Hsu et al. (16)																					
PSM cohort Whole cohort	Asia: 82% (Korea, Japan, China, Hong Kong, Taiwan); USA: 18%	Retrospective cohort study (19 centers from 6 countries or regions)	520 4,837	520 700	354 (68%) 3,328 (69%)	338 (65%) 456 (65%)	44 ± 0.5 51 ± 0.2	45 ± 0.6 46 ± 0.5	187 (36%) 1,537/4,653 (33%)	177 (34%) 208/617 (34%)	107 (21%) 1,344 (28%)^b^	105 (20%) 131 (19%)^b^	165 ± 16 200 ± 6	156 ± 14 147 ± 11	5.0 ± 0.1 5.5 ± 0.03	5.1 ± 0.1 5.0 ± 0.09	Yes Yes	60 (37–60) 44 (20–60)	39 (24–58) 34 (17–54)	1–6, 13, 14, 28, 29	(i) Age <18 yr; (ii) HCV, HDV, HIV; (iii) previous cancer, solid organ transplant, or immunosuppressant use; and (iv) HCC or death <1 yr of FU
Papatheodoridis et al. (20)																					
Whole cohort	Greece, Italy, Spain, Netherlands, and Turkey	PAGE-B cohort	772	1,163	538 (70%)	827 (71%)	52 ± 14	53 ± 13	110 (14%)	233 (20%)	166 (22%)	358 (31%)	54	36	5.4	3.3	^c^	91	90	1–6, 13^d^	(i) Age <16 yr; (ii) previous HCC, liver transplant; (iii) HCV, HDV, HIV; (iv) decompensated cirrhosis
Pol et al. (21)																					
Whole cohort	Europe, Africa, and Asia	ANRS CO22 Hepather cohort	1,143	1,515 (110 on both ETV & TDF)	n.a.	n.a.	n.a.	n.a.	n.a.	n.a.	n.a.	n.a.	n.a.	n.a.	n.a.	n.a.	n.a.	45 (26–53)		1, 2, 12, 13, 15, 29–31	(i) HCV and HDV
Kim et al. (13)																					
Whole cohort	USA	Administrative claims dataset	4,060	6,145	n.a.	n.a.	n.a.	n.a.	n.a.	n.a.	n.a.	n.a.	n.a.	n.a.	n.a.	n.a.	Yes	42	45	1, 2, 32, 33	(i) HCV, HDV, HIV and (ii) previous HCC or liver transplant or <6 mo after NA
Gordon et al. (22)																					
Whole cohort	USA (Asian and non-Asian)	Chronic Hepatitis Cohort Study	415	407	n.a.	n.a.	n.a.	n.a.	n.a.	n.a.	151 (18%)		n.a.	n.a.	n.a.	n.a.	^e^	36		1–3, 22, 30	(i) HIV and (ii) previous liver transplant
Ha et al. (23)																					
PSM cohort	South Korea	Retrospective cohort study (1 tertiary referral hospital)	298 921	298 419	181 (60.7%) 558	181 (60.1%) 266	48 ± 16 48 ± 15	48 ± 14 45 ± 16	161 (54.0%) 488	174 (58.4%) 261	39 (13.1%) 259	39 (13.1%) 39	93 ± 148 94 ± 120	98 ± 118 103 ± 132	6.4 ± 2.8 6.4 ± 2.3	6.3 ± 2.5 6.7 ± 2.6	Yes Yes	n.a. n.a.	n.a. n.a.	1–9, 11–14, 17, 27, 34	(i) Age <18 yr; (ii) duration of therapy <1 yr; (iii) previous HCC, liver transplant, or death or <6 mo after NA; and (iv) pretreatment HBV DNA <2,000 IU/mL
Whole cohort																					
Lee et al. (17)																					
Whole cohort	South Korea	Retrospective cohort study (1 tertiary referral hospital)	152	49	n.a.	n.a.	n.a.	n.a.	0	0	n.a.	n.a.	n.a.	n.a.	n.a.	n.a.	Yes	60 (range: 24–132)		1–3, 5–6	(i) HBeAg positivity, (ii) HCV and HIV, (iii) concomitant chronic liver diseases, (iv) decompensated cirrhosis, and (v) previous HCC
Oh et al. (24)																					
PSM cohort	South Korea	Retrospective cohort study (9 academic hospitals)	516 753	516 807	319 (61.8%) 480 (63.7%)	325 (63.0%) 503 (62.3%)	49 ± 13 49 ± 11	49 ± 9 46 ± 11	314 (60.9%) 451 (61.4%)	311 (60.3%) 484 (60.0%)	238 (46.1%) 315 (41.8%)	224 (43.4%) 310 (38.4%)	n.a. n.a.	n.a. n.a.	6.4 (5.4–7.5) 6.5 (5.4–7.6)	6.4 (5.4–7.5) 6.6 (5.5–7.7)	Yes Yes	56 (52–64) 59 (53–57)	58 (47–65) 56.4 (46–65)	1–5, 8–15, 22, 29, 35, 36	(i) HCV and HIV, (ii) duration of therapy <1 yr, and (iii) previous HCC or death <1 yr after NA
Whole cohort																					

Continue variables expressed as mean (±1 SD) or median (interquartile range).

AFP, alpha fetoprotein; ALT, alanine aminotransferase; APRI, AST/platelet ratio index; AST, aspartate aminotransferase; BMI, body mass index; CPS, Child-Pugh score; Cr, creatinine; CTP, Child-Turoctte-Pugh; DM, diabetes mellitus; ETV, entecavir; FIB-4, fibrosis-4 index; FU, follow-up; GGT, gamma-glutamyl transferase; HBeAg, hepatitis B e antigen; HBV, hepatitis B virus; HCC, hepatocellular carcinoma; HCV, hepatitis C virus; HDV, hepatitis D virus; INR, international normalized ratio; MELD, model for end-stage liver disease; n.a., not available; NA, nucleos(t)ide analog; Peg-IFN, pegylated-interferon; PS, propensity score; PSM, propensity score matched; PT, prothrombin time; RRT, renal replacement therapy; SES, socioeconomic status; TDF, tenofovir.

a Decompensated cirrhosis: ETV group 421 (4%), TDF group 388 (4%).

b CPS B/C: ETV group 136 (3%), TDF group 29 (4%).

c Both treatment naive and experienced.

d Not adjusted for HBeAg, previous treatment use, ALT, HBV DNA despite significant difference between ETV and TDF groups.

e One hundred sixty-four (20%): only treatment-naive patients were included for analysis in current meta-analysis.

1, age; 2, sex; 3, cirrhosis; 4, HBeAg; 5, HBV DNA; 6, ALT; 7, AST; 8, albumin; 9, bilirubin; 10, creatinine; 11, AFP; 12, INR or PT; 13, platelet; 14, DM; 15, hypertension; 16, smoking; 17, drinking; 18, BMI; 19, SES; 20, healthcare level; 21, APRI; 22, FIB-4; 23, CPS; 24, varix; 25, GGT; 26, RRT; 27, calendar year of treatment initiation; 28, country; 29, hepatic decompensation; 30, ethnicity; 31, fibrosis stage; 32, health conditions; 33, weighting based on treatment PS; 34, HBsAg titer; 35, CTP; 36, MELD.

A total of 85,008 patients with CHB (ETV: 56,346; TDF: 28,662) were included. Male proportion was 76.2%, median age was 49 years (IQR: 47–51; range: 36–53), HBeAg was 43.7%, cirrhosis was 22.5%, median ALT was 94 (IQR: 46–129; range: 32–200), and median HBV DNA was 5.9 log_10_IU/mL (IQR: 5.4–6.4; range: 3.3–6.7). All studies recruited treatment-naive patients, except for the study by Papatheodoridis et al. Although Gordon et al. also recruited both treatment-naive and experienced patients, they provided aHR after excluding treatment-experienced patients (and this figure was used for the primary analysis). Eight studies provided details on the follow-up time of both the ETV and TDF groups. Only 3 studies showed a minimal difference in follow-up between the 2 groups (ranging from 1 to 3 months), whereas the other studies showed much shorter follow-up time for TDF vs ETV groups (ranging from 10 to 33 months). Five studies did not adjust for HBV DNA. Importantly, Choi et al. also did not adjust for liver function parameters (including bilirubin and albumin) and platelet count.

### Meta-analysis

**Figure 2.**
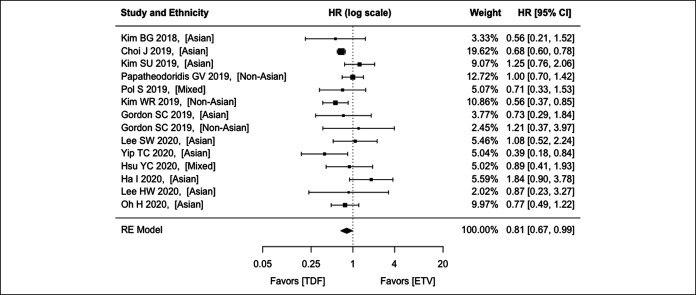
Comparison between ETV and TDF on hepatocellular carcinoma preventive effect among patients with CHB using the REs model. CHB, chronic hepatitis B; CI, confidence interval; ETV, entecavir; HR, hazard ratio; RE, random effect; TDF, tenofovir disoproxil fumarate.

Of the 13 studies included, 8 reported the aHR of HCC with TDF compared with ETV by both PS matching and multivariable analysis, and 5 by multivariable analysis only. There was no significant heterogeneity among the studies (*P* = 0.066; *I*^2^ = 43.4%). A random effects model yields an aHR of 0.81 (95% CI: 0.67–0.99; *P* = 0.041) (Figure 2). A fixed effects model yields an aHR of 0.74 (95% CI: 0.67–0.82; *P* < 0.001). Funnel plot did not suggest publication bias (Egger test: *P* = 0.624) (see eFigure 1, Supplementary Digital Content 1, http://links.lww.com/CTG/A384).

### Subgroup analysis

#### Cirrhosis

**Figure 3.**
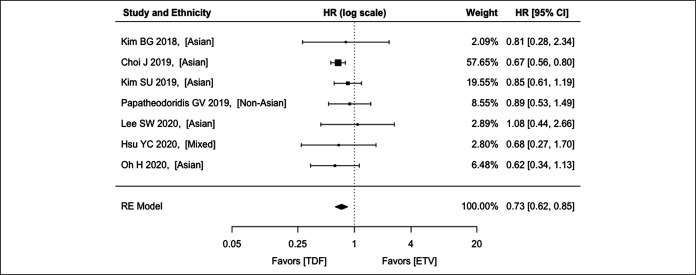
Comparison between ETV and TDF on hepatocellular carcinoma preventive effect among patients with CHB with cirrhosis using the REs model. CHB, chronic hepatitis B; CI, confidence interval; ETV, entecavir; HR, hazard ratio; RE, random effect; TDF, tenofovir disoproxil fumarate.

For cirrhosis, 7 studies (5 recruiting Asians, 1 recruiting non-Asians, and 1 recruiting both) were analyzed, with an aHR of 0.73 (95% CI: 0.62–0.85; *P* < 0.001) (Figure 3). There was no significant heterogeneity among the studies (*P* = 0.770; *I*^2^ = 4.2%)

**Figure 4.**
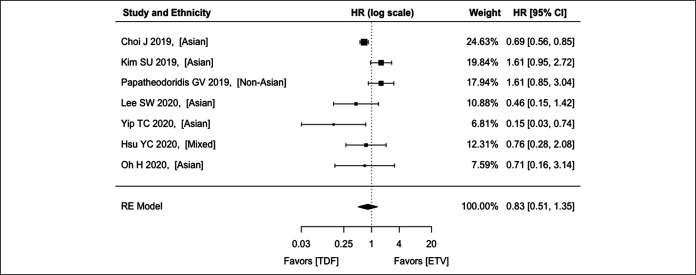
Comparison between ETV and TDF on hepatocellular carcinoma preventive effect among patients with CHB without cirrhosis using the REs model. CHB, chronic hepatitis B; CI, confidence interval; ETV, entecavir; HR, hazard ratio; RE, random effect; TDF, tenofovir disoproxil fumarate.

For noncirrhosis, 7 studies (5 recruiting Asians, 1 recruiting non-Asians, and 1 recruiting both) were analyzed, with an aHR of 0.83 (95% CI: 0.51–1.35; *P* = 0.457) (Figure 4). There was significant heterogeneity among the studies (*P* = 0.005; *I*^2^ = 68.8%).

#### Ethnicity

One study Pol et al. involving a heterogeneous population of Asian and non-Asian patients was excluded from this subgroup analysis. Although Hsu et al. and Gordon et al. recruited both Asians and non-Asians, the aHRs were available for Asian and non-Asian patients separately.

For the Asian population, 10 studies were analyzed, with an aHR of 0.82 (95% CI: 0.63–1.06; *P* = 0.067) (see eFigure 2, Supplementary Digital Content 1, http://links.lww.com/CTG/A384). There was no significant heterogeneity among the studies (*P* = 0.067; *I*^2^ = 46.8%).

For the non-Asian population, 4 studies were analyzed, with an aHR of 0.80 (95% CI: 0.53–1.22; *P* = 0.301) (see eFigure 3, Supplementary Digital Content 1, http://links.lww.com/CTG/A384). There was no significant heterogeneity among the studies (*P* = 0.195; *I*^2^ = 43.6%).

#### Study design

Three retrospective cohort studies using electronic databases were analyzed, with an aHR of 0.63 (95% CI: 0.51–0.78; *P* < 0.001) (see eFigure 4, Supplementary Digital Content 1, http://links.lww.com/CTG/A384). There was no significant heterogeneity among the studies (*P* = 0.276; *I*^2^ = 22.3%).

Eleven retrospective cohort studies using clinical records were analyzed, with an aHR of 0.97 (95% CI: 0.80–1.18; *P* = 0.787) (see eFigure 5, Supplementary Digital Content 1, http://links.lww.com/CTG/A384). There was no significant heterogeneity among the studies (*P* = 0.678; *I*^2^ = 0%).

### Sensitivity analysis

eTable 2 (see Supplementary Digital Content 1, http://links.lww.com/CTG/A384) shows the leave-one-out sensitivity analysis using random effects model. Excluding the study by Choi et al. (accounting for largest weight in the pooled analysis) or Kim et al. has the largest effect on the pooled HR and *P* value. The pooled HR increased from 0.81 to 0.85, and the *P* values were >0.10. Contrarily, excluding the study by Kim et al. or Ha et al. caused a change of the pooled HR from 0.81 to 0.77.

Pooling individual aHR derived by multivariable analysis yields an aHR of 0.80 (95% CI: 0.67–0.95; *P* = 0.010) (see eFigure 6, Supplementary Digital Content 1, http://links.lww.com/CTG/A384). There was significant heterogeneity among the studies (*P* = 0.027; *I*^2^ = 46.5%).

The subgroup analysis performed by excluding the study by Choi et al. shows that the beneficial effect did not exist for various subgroup, except for limiting analysis to studies using electronic databases (HR: 0.52, 95% CI: 0.36–0.75; *P* < 0.001) (see eTable 3, Supplementary Digital Content 1, http://links.lww.com/CTG/A384).

Sensitivity analysis was also performed by excluding the study by Papatheodoridis et al., which recruited both treatment-naive and experienced patients. There was no significant heterogeneity among the studies (*P* = 0.104; *I*^2^ = 42.0%). A random effects model yields a pooled aHR of 0.79 (95% CI: 0.64–0.98; *P* = 0.033).

## DISCUSSION

In this meta-analysis of 11 studies, we found that TDF was associated with a 23% lower HCC risk compared with ETV in patients with CHB. On the subgroup analysis, this beneficial effect persisted in patients who have cirrhosis and from retrospective cohort studies that used electronic data sets.

There are 3 possible explanations for this observation. First, nucleotides, but not NAs, have been found to induce higher levels of serum interferon lamda-3 (32) that has potent antitumor activity in murine models of cancer and inhibits HBsAg production (33). A RCT also showed that TDF more effectively reduced the HBsAg level than ETV (34). Second, TDF may have a better antiviral effect than ETV, with a Bayesian meta-analysis showing that TDF leads to a higher rate of serum HBV DNA undetectability at 1 year of treatment (7). Third, although both NAs are considered to have high genetic barriers to resistance, TDF is probably superior. The resistance rate of ETV was 1.2% after 5 years of treatment (1), although no drug resistance was documented after 8 years of TDF (35).

To date, whether TDF is superior to ETV in HCC prevention remains controversial, with some studies showing favoring TDF, whereas others showing no difference between these 2 NAs. This controversy may exist because of several reasons. The most important one is the inadequate sample size of individual studies. Of the 8 studies reporting no significant difference, 5 actually showed a trend toward better cancer preventive effect for TDF vs ETV (Figure 2). The sample size of these 5 studies ranged from 822 to 2,768 (Table 1) that may be under-powered to detect a statistically significant difference.

We also showed that TDF is better than ETV in reducing HCC risk in cirrhotic patients only, but not noncirrhotic patients, although this should be interpreted with caution because of underpower from the subgroup analysis. Another source of controversy could stem from ethnicity of the study population. We show that the beneficial effect did not exist in non-Asians and was of borderline significance for Asians. This observation was well illustrated in the study by Hsu et al. (a median follow-up of 60 months) (16), in which the Kaplan-Meier plot shows that HCC risk started to diverge between ETV and TDF groups among Asians at the second year of follow-up (logrank *P* = 0.05), but not among non-Asians (log-rank *P* = 0.87). Gordon et al. also showed a numerical difference in effect estimates between the 2 ethnicities (aHR in Asians: 0.73; non-Asians: 1.21). Although no statistical significance was detected in the stratified analysis, this could be because of underpower (517 Asians only). Whether beneficial effect of TDF over ETV in Asians requires further studies with a large sample size to confirm because the current meta-analysis shows that it was of borderline significance (*P* = 0.067). We hypothesize that Asians, whether residing in Asia or in other countries, contracted CHB virus early in life from carrier parents, whereas Whites usually get infected during adulthood, which may lead to a lower HCC risk (36).

Different study designs also likely contributed to the controversy. There was a statistically significant difference for the retrospective cohort studies using electronic data sets but not for prospective/retrospective cohort studies using data from clinical records. The prevalence of nonadvanced cirrhosis may be underestimated in retrospective cohort studies using electronic data sets, identification of which was dependent on the diagnosis code entry, and transient elastography might not be a routine. The management strategy in liver function test, HBV DNA monitoring, and HCC surveillance was not unified, unlike in prospective cohort settings. HBV DNA suppression and development of resistance to ETV were unlikely to be fully adjusted for. For example, Choi et al. (12) had fewer PS-matched parameters with no HBV DNA and some liver function parameters including bilirubin, albumin, platelet counts, and prothrombin time in the nationwide cohort.

There are several strengths of our study. Our study addressed the major limitations of 2 recent meta-analyses (26,37) that did not include the more recent high-quality studies and included studies that reported either number of events or crude/unadjusted effect estimate. Owing to the later introduction of TDF, physicians may preferentially prescribe ETV to patients with more severe liver disease because of the longer available drug data. Older patients, those with renal impairment and metabolic comorbidities associated with renal disease (e.g., diabetes and hypertension) may be given ETV because long-term use of TDF is associated with renal impairment. Failure to consider this indication bias (i.e., TDF users are generally healthier and have milder liver disease) by adjusting for the liver disease severity, comorbidities including renal impairment, and age will lead to serious bias, favoring TDF for HCC risk reduction. Second, only studies that provided aHR from the PS methodology/multivariable analysis were included, minimizing indication/selection bias. Third, bias resulting from the marked difference in the follow-up duration between the 2 groups could be partially addressed by pooling HR instead of OR. Fourth, subgroup analysis according to cirrhosis, ethnicity, and study design explains the source of controversy among different studies.

There are some limitations in this study. The most important one is disparity in the follow-up periods between the 2 groups even after PS matching in some of the included studies (the ETV group had longer follow-up than the TDF group—a difference of up to 33 months). This differential follow-up time may spuriously favor a better preventive effect of TDF over ETV because HCC takes years to develop. Second, residual/unmeasured confounding factors are still possible despite PS methodology or multivariable analysis. For instance, drug compliance and HCC surveillance program data could not be captured in electronic data sets. Third, most of the studies had a follow-up time of up to 5 years only. Longer-term studies of >10 years would help to draw more definitive conclusions. Fourth, beneficial effect of TDF over ETV should be interpreted with caution because the statistically significant association was dominated by the study by Choi et al. Sensitivity analysis showed that the association was no longer statistically significant after excluding this study, except for limiting analysis to studies using electronic databases. Fifth, HBV genotype, a known risk factor for HCC with genotype C conferring higher risk than others (38), was not analyzed in these studies. Finally, studies did not adjust for aspirin (39), statins (40), and metformin (41) that lower HCC risk and are more frequently prescribed in ETV users because of associated medical comorbidities. Failure to adjust for these factors may attenuate potential chemopreventive effect of TDF when compared with ETV.

TDF was associated with a lower HCC risk compared with ETV in patients with CHB, particularly cirrhotic patients. Further prospective studies with larger sample size and longer follow-up period to identify specific subgroups (according to cirrhosis, ethnicity, and HBeAg seropositivity) that benefit most from TDF should be identified.

## CONFLICTS OF INTEREST

**Guarantor of the article:** Ching Lung Lai, MD.

**Specific author contributions:** K.S.C.: involved with study concept and design, literature search, analysis and interpretation of data, drafting of the manuscript, and approval of the final version of the manuscript. L.Y.M.: involved with literature search and approval of the final version of the manuscript. S.H.L., W.K.S.: involved with interpretation of data and approval of the final version of the manuscript. M.F.Y., C.L.L.: involved with the study concept and design, analysis and interpretation of data, drafting of the manuscript, critical revision of the manuscript for important intellectual content, study supervision, and approval of the final version of the manuscript.

**Financial support:** None to report.

**Potential competing interests:** C.L.L. has given sponsored lectures for Bristol Myers Squibb and Gilead Sciences. M.F.Y. received speaker honoraria from Bristol Myers Squibb and Gilead Sciences and unrestricted hepatitis B related research grants from Bristol Myers Squibb and Gilead Sciences. There are no competing interests for the other authors.

**Study Highlights**

**WHAT IS KNOWN**

✓ Both entecavir and tenofovir can reduce hepatocellular carcinoma (HCC) risk among chronic hepatitis B (CHB) patients.

**WHAT IS NEW HERE**

✓ Tenofovir disoproxil fumarate (TDF) is associated with a lower HCC risk among CHB patients in this meta-analysis.
✓ This beneficial effect is most prominent among patients with cirrhosis.

## Supplementary Material

SUPPLEMENTARY MATERIAL
