# Supplementary material for: Entecavir vs Tenofovir in Hepatocellular Carcinoma Prevention in Chronic Hepatitis B Infection: A Systematic Review and Meta-Analysis
Source: Clin Transl Gastroenterol. 2020 Oct 6;11(10):e00236. doi: 10.14309/ctg.0000000000000236 (PMC7544163; doi:10.14309/ctg.0000000000000236)
Supplement: SUPPLEMENTARY MATERIAL [file ct9-11-e00236-s001.pdf]

ENTECAVIR VS TENOFOVIR IN HEPATOCELLULAR CARCINOMA PREVENTION IN CHRONIC HEPATITIS B INFECTION: A SYSTEMATIC REVIEW AND META-ANALYSIS

Ka Shing Cheung, MBBS, MPH;[1,2] Lung Yi Mak, MBBS;[1] Sze Hang Liu, MBChB;[1] Ho Ming Cheng, PhD;[1] Wai Kay Seto, MD;[1,2] Man Fung Yuen, DSc;[1] Ching Lung Lai, MD[1]

[1]Department of Medicine, The University of Hong Kong, Queen Mary Hospital, Hong Kong

[2]Department of Medicine, The University of Hong Kong-Shenzhen Hospital, Shenzhen, China

Keywords: nucleotide analogue, nucleoside analogue, liver cancer, HBV

Corresponding author

Department of Medicine, Queen Mary Hospital, 102 Pokfulam Road, Hong Kong

Email: hrmelcl@hku.hk

Fax: +852 2816 2863

Phone: + 852 2255 4252

Guarantor of the article: Prof. Ching Lung Lai

Tables of Contents

Search details for Pubmed, Embase and Cochrane Library	pages 3-4
eTable 1. Quality assessment according to Newcastle-Ottawa quality assessment scale (NOS)	pages 5
eTable 2. Leave-one-out sensitivity analysis	page 6
eTable 3. Pooled HR by excluding the study by Choi J et al using random effects model	page 7
Figure Legend	pages 8-9
eFigure 1. Funnel plot for detecting publication bias	page 10
eFigure 2. Comparison between ETV and TDF on hepatocellular carcinoma preventive effect among Asian CHB patients (random effects model)	page 11
eFigure 3. Comparison between ETV and TDF on hepatocellular carcinoma preventive effect among non-Asian CHB patients (random effects model)	page 12
eFigure 4. Comparison between ETV and TDF on hepatocellular carcinoma preventive effect among studies using electronic databases (random effects model)	page 13
eFigure 5. Comparison between ETV and TDF on hepatocellular carcinoma preventive effect among studies using clinical records (random effects model)	page 14
eFigure 6. Comparison between ETV and TDF on hepatocellular carcinoma preventive effect among CHB patients by pooling results from multivariable analysis (random effects model)	page 15
PRIMSA checklist	pages 16-18

Search details for Pubmed, Embase and Cochrane Library

Pubmed

(("entecavir"[Supplementary Concept] OR "entecavir"[All Fields]) AND ("tenofovir"[MeSH Terms] OR "tenofovir"[All Fields])) AND (("carcinoma, hepatocellular"[MeSH Terms] OR ("carcinoma"[All Fields] AND "hepatocellular"[All Fields]) OR "hepatocellular carcinoma"[All Fields] OR ("liver"[All Fields] AND "cell"[All Fields] AND "carcinoma"[All Fields]) OR "liver cell carcinoma"[All Fields]) OR ("liver neoplasms"[MeSH Terms] OR ("liver"[All Fields] AND "neoplasms"[All Fields]) OR "liver neoplasms"[All Fields] OR ("liver"[All Fields] AND "tumor"[All Fields]) OR "liver tumor"[All Fields]) OR ("liver neoplasms"[MeSH Terms] OR ("liver"[All Fields] AND "neoplasms"[All Fields]) OR "liver neoplasms"[All Fields] OR ("hepatic"[All Fields] AND "neoplasm"[All Fields]) OR "hepatic neoplasm"[All Fields]) OR ("carcinoma, hepatocellular"[MeSH Terms] OR ("carcinoma"[All Fields] AND "hepatocellular"[All Fields]) OR "hepatocellular carcinoma"[All Fields] OR ("hepatocellular"[All Fields] AND "carcinoma"[All Fields])) OR ("liver neoplasms"[MeSH Terms] OR ("liver"[All Fields] AND "neoplasms"[All Fields]) OR "liver neoplasms"[All Fields] OR ("liver"[All Fields] AND "cancer"[All Fields]) OR "liver cancer"[All Fields]) OR ("carcinoma, hepatocellular"[MeSH Terms] OR ("carcinoma"[All Fields] AND "hepatocellular"[All Fields]) OR "hepatocellular carcinoma"[All Fields] OR "hepatoma"[All Fields]) OR ("carcinoma, hepatocellular"[MeSH Terms] OR ("carcinoma"[All Fields] AND "hepatocellular"[All Fields]) OR "hepatocellular carcinoma"[All Fields] OR "hepatocarcinoma"[All Fields]) OR ("liver neoplasms"[MeSH Terms] OR ("liver"[All Fields] AND "neoplasms"[All Fields]) OR "liver neoplasms"[All Fields] OR ("liver"[All Fields] AND "neoplasm"[All Fields]) OR "liver neoplasm"[All Fields]))

Embase

1. entecavir

2. tenofovir

3. 1 AND 2

4. liver cell carcinoma

5. liver tumor

6. hepatic neoplasm

7. hepatocellular carcinoma

8. liver cancer

9. hepatoma

10. hepatocarcinoma

11. liver neoplasm

12. 4 OR 5 OR 6 OR 7 OR 8 OR 9 OR 10 OR 11

13. 3 AND 12

Cochrane Library

(entecavir or tenofovir) AND (liver cell carcinoma or liver tumor or hepatic neoplasm or hepatocellular carcinoma or liver cancer or hepatoma or hepatocarcinoma or liver neoplasm)

eTable 1. Quality assessment according to Newcastle-Ottawa quality assessment scale (NOS)

	References	Kim BG 2018	Choi J 2019	Kim SU 2019	Lee SW 2019	Yip TCF 2019	Hsu YC 2019	Paptheod oridis GV 2019	Pols S 2019	Kim WR 2019	Gordon SC 2019	Ha I 2020	Lee HW 2020	Oh H 2020
**Selection**	Representativeness of exposed cohort	1	1	1	1	1	1	1	1	1	1	1	1	1
	Selection of non-exposed cohort	1	1	1	1	1	1	1	1	1	1	1	1	1
	Ascertainment of exposure	1	1	1	1	1	1	1	1	1	1	1	1	1
	Demonstration that outcome of interest was not present at the start of study	1	1	1	1	1	1	1	Unclear	1	Unclear	1	1	1
**Comparability**	Controls for age or gender	1	1	1	1	1	1	1	1	1	1	1	1	1
	Controls for additional factor	1	1	1	1	1	1	1	1	1	1	1	1	1
**Outcome**	Assessment of outcome	1	1	1	1	1	1	1	1	1	1	1	1	1
	Follow-up long enough for outcomes to occur	1	1	1	1	1	1	1	1	1	1	1	1	1
	Adequacy of follow-up of cohort	1	1	1	1	1	1	1	1	1	1	1	1	1
	Total	9	9	9	9	9	9	9	8	9	8	9	9	9

eTable 2. Leave-one-out sensitivity analysis using random effects model

Study*	Ethnicity	Pooled HR	95% CI	p-value
Kim BG 2018	Asian	0.83	0.67 – 1.01	0.067
Choi J 2019	Asian	0.85	0.68 – 1.07	0.158
Kim SU 2019	Asian	0.77	0.64 – 0.93	0.008
Papatheodoridis GV 2019	Non-Asian	0.79	0.64 – 0.98	0.033
Pol S 2019	Mixed	0.82	0.67 – 1.01	0.068
Kim WR 2019	Non-Asian	0.85	0.69 – 1.04	0.117
Gordon SC 2019	Asian	0.82	0.67 – 1.01	0.059
Gordon SC 2019	Non-Asian	0.81	0.66 – 0.99	0.036
Lee SW 2020	Asian	0.80	0.65 – 0.98	0.035
Yip TC 2020	Asian	0.84	0.69 – 1.03	0.089
Hsu YC 2020	Mixed	0.81	0.66 – 1.00	0.051
Ha I 2020	Asia	0.77	0.64 – 0.92	0.004
Lee HW 2020	Asian	0.81	0.66 – 1.00	0.045
Oh H 2020	Asian	0.82	0.66 – 1.03	0.085

* Individual study in each row was excluded to calculate the pooled HR to assess impact of single study on the pooled effect estimate

Abbreviations: HR, hazard ratio; 95% CI, 95% confidence interval

eTable 3. Pooled HR by excluding the study by Choi J et al using random effects model

	Pooled HR	95% CI	p-value
Main result	0.85	0.68 – 1.07	0.158
Cirrhosis	0.82	0.65 – 1.03	0.087
Non-cirrhosis	0.84	0.45 – 1.59	0.592
Asian population	0.87	0.64 – 1.19	0.386
Non-Asian population	0.80	0.53 – 1.22	0.301
Studies using electronic databases	0.52	0.36 – 0.75	<0.001
Studies using clinical records	0.97	0.80 – 1.18	0.787

Abbreviations: HR, hazard ratio; 95% CI: 95% confidence interval

Figure legend

eFigure 1. Funnel plot for detecting publication bias

eFigure 2. Comparison between ETV and TDF on hepatocellular carcinoma preventive effect among Asian CHB patients (random effects model)

Abbreviations: ETV, entecavir; TDF, tenofovir disoproxil fumarate; CHB, chronic hepatitis B; HR, hazard ratio; RE, random effects

eFigure 3. Comparison between ETV and TDF on hepatocellular carcinoma preventive effect among non-Asian CHB patients (random effects model)

Abbreviations: ETV, entecavir; TDF, tenofovir disoproxil fumarate; CHB, chronic hepatitis B; HR, hazard ratio; RE, random effects

eFigure 4. Comparison between ETV and TDF on hepatocellular carcinoma preventive effect among studies using electronic databases (random effects model)

Abbreviations: ETV, entecavir; TDF, tenofovir disoproxil fumarate; HR, hazard ratio; RE, random effects

eFigure 5. Comparison between ETV and TDF on hepatocellular carcinoma preventive effect among studies using clinical records (random effects model)

Abbreviations: ETV, entecavir; TDF, tenofovir disoproxil fumarate; HR, hazard ratio; RE, random effects

eFigure 6. Comparison between ETV and TDF on hepatocellular carcinoma preventive effect among CHB patients by pooling results from multivariable analysis (random effects model)

Abbreviations: ETV, entecavir; TDF, tenofovir disoproxil fumarate; CHB, chronic hepatitis B; HR, hazard ratio; RE, random effects

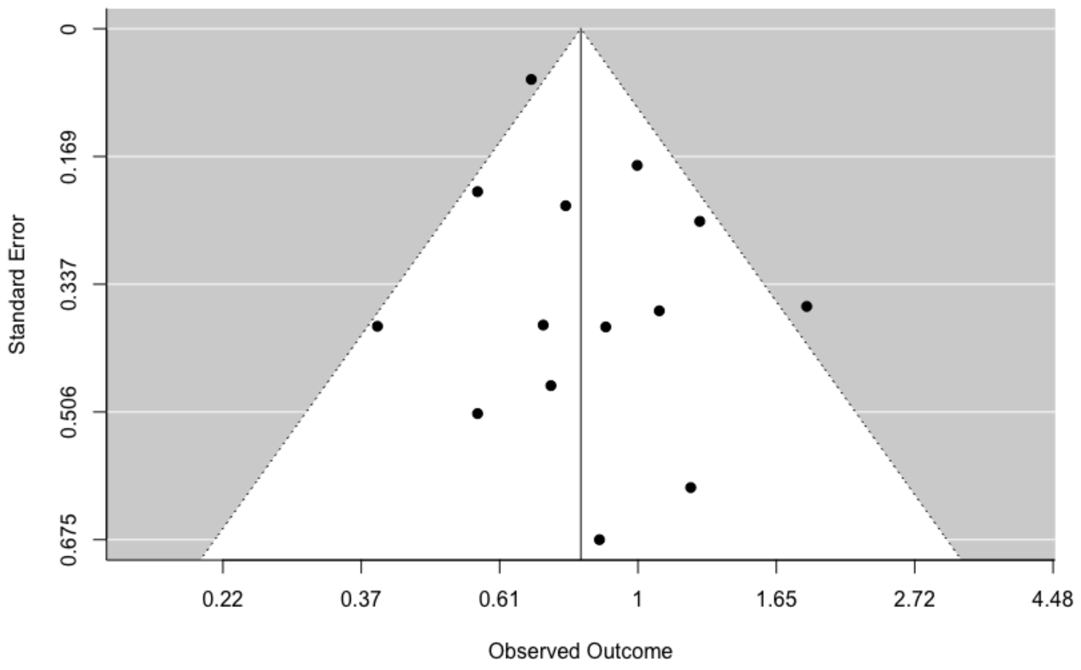

eFigure 1. Funnel plot for detecting publication bias

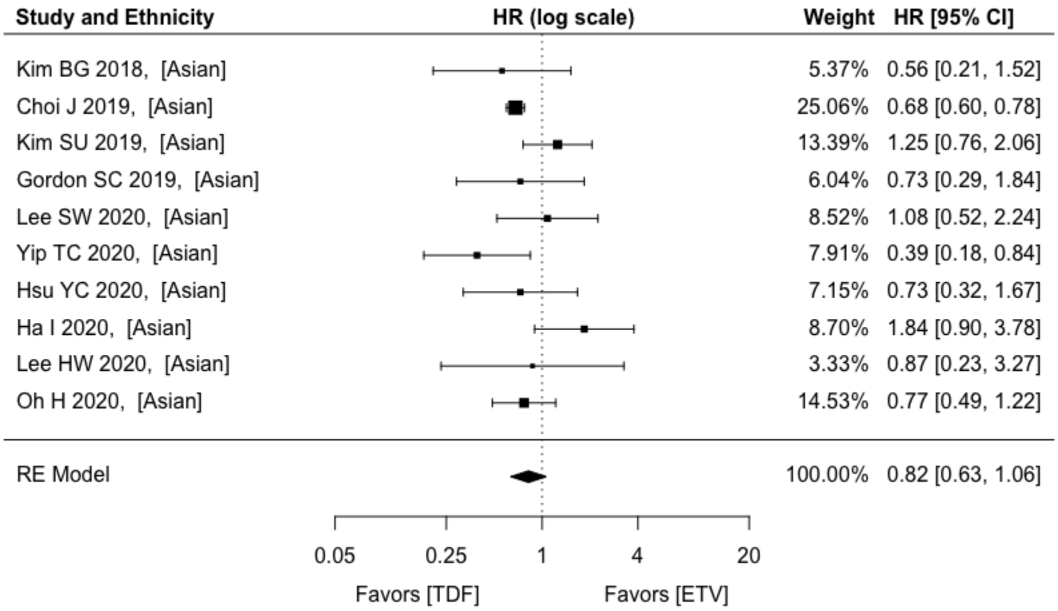

eFigure 2. Comparison between ETV and TDF on hepatocellular carcinoma preventive effect among Asian CHB patients (random effects model)

Abbreviations: ETV, entecavir; TDF, tenofovir disoproxil fumarate; CHB, chronic hepatitis B; HR, hazard ratio; RE, random effects

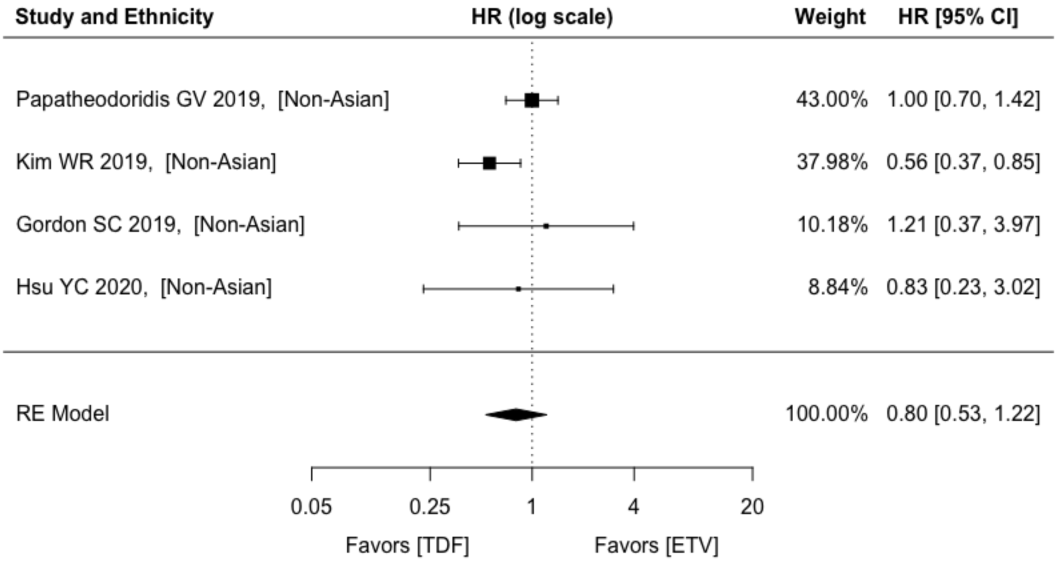

eFigure 3. Comparison between ETV and TDF on hepatocellular carcinoma preventive effect among non-Asian CHB patients (random effects model)

Abbreviations: ETV, entecavir; TDF, tenofovir disoproxil fumarate; CHB, chronic hepatitis B; HR, hazard ratio; RE, random effects

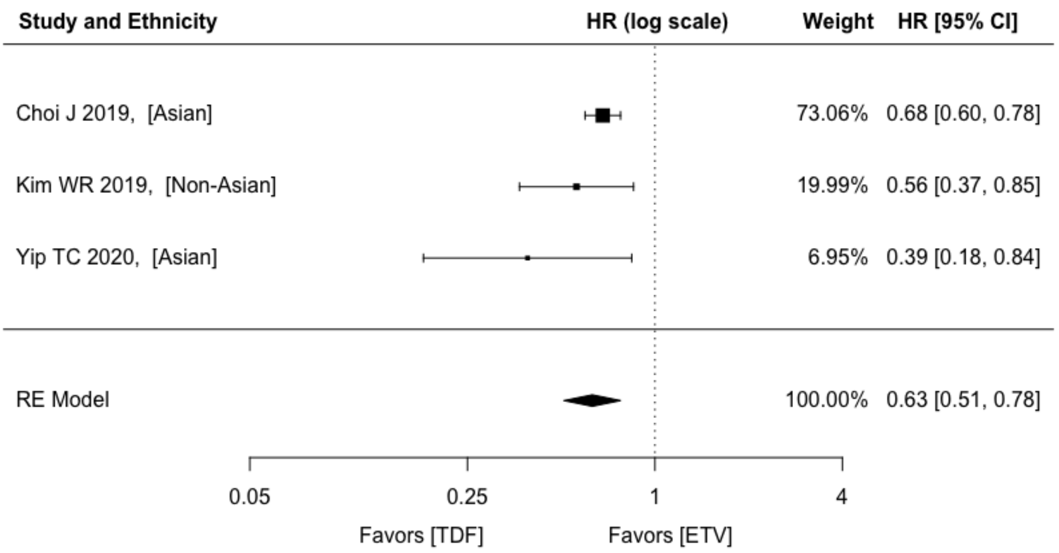

eFigure 4. Comparison between ETV and TDF on hepatocellular carcinoma preventive effect among studies using electronic databases (random effects model)

Abbreviations: ETV, entecavir; TDF, tenofovir disoproxil fumarate; HR, hazard ratio; RE, random effects

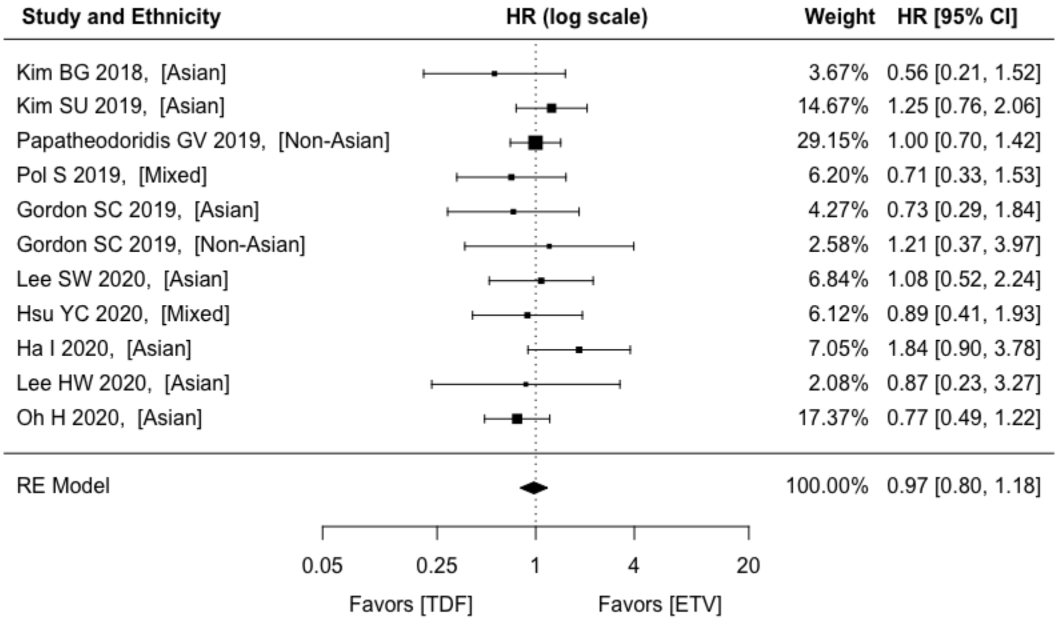

eFigure 5. Comparison between ETV and TDF on hepatocellular carcinoma preventive effect among studies using clinical records (random effects model)

Abbreviations: ETV, entecavir; TDF, tenofovir disoproxil fumarate; HR, hazard ratio; RE, random effects

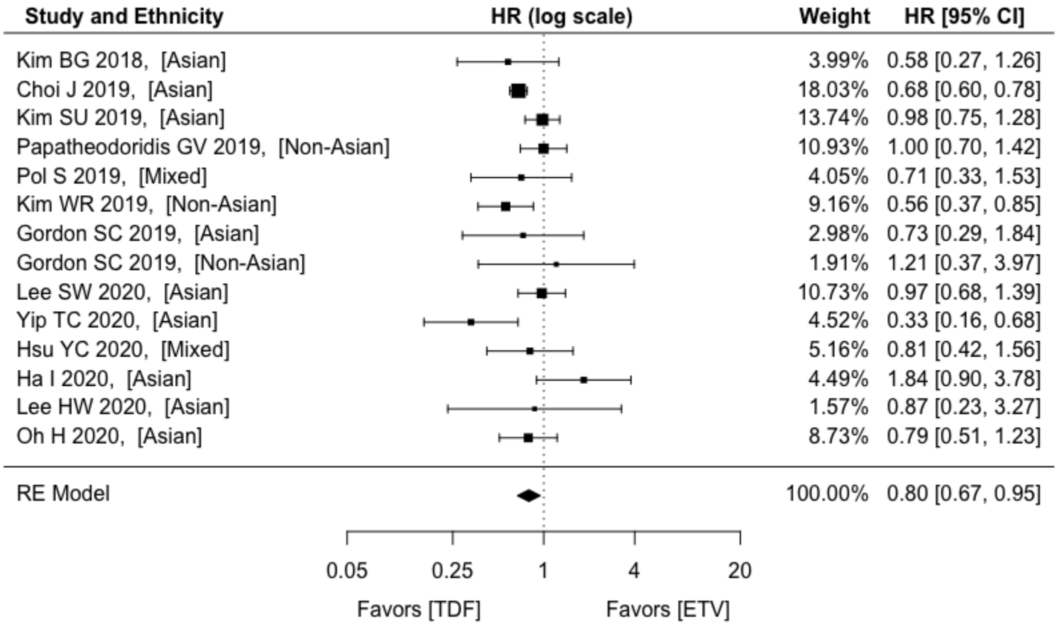

eFigure 6. Comparison between ETV and TDF on hepatocellular carcinoma preventive effect among CHB patients by pooling results from multivariable analysis (random effects model)

Abbreviations: ETV, entecavir; TDF, tenofovir disoproxil fumarate; CHB, chronic hepatitis B; HR, hazard ratio; RE, random effects

PRISMA 2009 Checklist

Section/topic	#	Checklist item	Reported on page #
**TITLE**			
Title	1	Identify the report as a systematic review, meta-analysis, or both.	1
**ABSTRACT**			
Structured summary	2	Provide a structured summary including, as applicable: background; objectives; data sources; study eligibility criteria, participants, and interventions; study appraisal and synthesis methods; results; limitations; conclusions and implications of key findings; systematic review registration number.	3, 4
**INTRODUCTION**			
Rationale	3	Describe the rationale for the review in the context of what is already known.	5,6
Objectives	4	Provide an explicit statement of questions being addressed with reference to participants, interventions, comparisons, outcomes, and study design (PICOS).	5,6
**METHODS**			
Protocol and registration	5	Indicate if a review protocol exists, if and where it can be accessed (e.g., Web address), and, if available, provide registration information including registration number.	7
Eligibility criteria	6	Specify study characteristics (e.g., PICOS, length of follow-up) and report characteristics (e.g., years considered, language, publication status) used as criteria for eligibility, giving rationale.	7,8
Information sources	7	Describe all information sources (e.g., databases with dates of coverage, contact with study authors to identify additional studies) in the search and date last searched.	7
Search	8	Present full electronic search strategy for at least one database, including any limits used, such that it could be repeated.	Supplementary material
Study selection	9	State the process for selecting studies (i.e., screening, eligibility, included in systematic review, and, if applicable, included in the meta-analysis).	7, Figure 1
Data collection process	10	Describe method of data extraction from reports (e.g., piloted forms, independently, in duplicate) and any processes for obtaining and confirming data from investigators.	7,8
Data items	11	List and define all variables for which data were sought (e.g., PICOS, funding sources) and any assumptions and simplifications made.	7,8
Risk of bias in individual studies	12	Describe methods used for assessing risk of bias of individual studies (including specification of whether this was done at the study or outcome level), and how this information is to be used in any data synthesis.	8,9
Summary measures	13	State the principal summary measures (e.g., risk ratio, difference in means).	8,9

PRISMA 2009 Checklist

Synthesis of results	14	Describe the methods of handling data and combining results of studies, if done, including measures of consistency (e.g., I^2) for each meta-analysis.	8,9

Section/topic	#	Checklist item	Reported on page #
Risk of bias across studies	15	Specify any assessment of risk of bias that may affect the cumulative evidence (e.g., publication bias, selective reporting within studies).	9
Additional analyses	16	Describe methods of additional analyses (e.g., sensitivity or subgroup analyses, meta-regression), if done, indicating which were pre-specified.	9
**RESULTS**			
Study selection	17	Give numbers of studies screened, assessed for eligibility, and included in the review, with reasons for exclusions at each stage, ideally with a flow diagram.	11, Figure 1
Study characteristics	18	For each study, present characteristics for which data were extracted (e.g., study size, PICOS, follow-up period) and provide the citations.	11, 12, Table 1
Risk of bias within studies	19	Present data on risk of bias of each study and, if available, any outcome level assessment (see item 12).	12,13,14
Results of individual studies	20	For all outcomes considered (benefits or harms), present, for each study: (a) simple summary data for each intervention group (b) effect estimates and confidence intervals, ideally with a forest plot.	Figures 2-4, eFigures2-6
Synthesis of results	21	Present results of each meta-analysis done, including confidence intervals and measures of consistency.	12,13,14
Risk of bias across studies	22	Present results of any assessment of risk of bias across studies (see Item 15).	12,13,14
Additional analysis	23	Give results of additional analyses, if done (e.g., sensitivity or subgroup analyses, meta-regression [see Item 16]).	12,13,14
**DISCUSSION**			
Summary of evidence	24	Summarize the main findings including the strength of evidence for each main outcome; consider their relevance to key groups (e.g., healthcare providers, users, and policy makers).	15,16,17
Limitations	25	Discuss limitations at study and outcome level (e.g., risk of bias), and at review-level (e.g., incomplete retrieval of identified research, reporting bias).	17,18
Conclusions	26	Provide a general interpretation of the results in the context of other evidence, and implications for future research.	18
**FUNDING**			

Funding	27	Describe sources of funding for the systematic review and other support (e.g., supply of data); role of funders for the systematic review.	2

From: Moher D, Liberati A, Tetzlaff J, Altman DG, The PRISMA Group (2009). Preferred Reporting Items for Systematic Reviews and Meta-Analyses: The PRISMA Statement. PLoS Med 6(7): e1000097. doi:10.1371/journal.pmed1000097

For more information, visit: **www.prisma-statement.org**.
